# VSEAMS: a pipeline for variant set enrichment analysis using summary GWAS data identifies *IKZF3*, *BATF* and *ESRRA* as key transcription factors in type 1 diabetes

**DOI:** 10.1093/bioinformatics/btu571

**Published:** 2014-08-27

**Authors:** Oliver S. Burren, Hui Guo, Chris Wallace

**Affiliations:** ^1^Department of Medical Genetics, JDRF/Wellcome Trust Diabetes and Inflammation Laboratory, NIHR Cambridge Biomedical Research Centre, Cambridge Institute for Medical Research, University of Cambridge, Wellcome Trust/MRC Building, Cambridge Biomedical Campus, Cambridge, CB2 0XY, UK and ^2^MRC Biostatistics Unit, Cambridge Institute of Public Health, Forvie Site, Robinson Way, Cambridge Biomedical Campus, Cambridge, CB2 0SR, UK

## Abstract

**Motivation**: Genome-wide association studies (GWAS) have identified many loci implicated in disease susceptibility. Integration of GWAS summary statistics (*P*-values) and functional genomic datasets should help to elucidate mechanisms.

**Results**: We extended a non-parametric SNP set enrichment method to test for enrichment of GWAS signals in functionally defined loci to a situation where only GWAS *P*-values are available. The approach is implemented in VSEAMS, a freely available software pipeline. We use VSEAMS to identify enrichment of type 1 diabetes (T1D) GWAS associations near genes that are targets for the transcription factors *IKZF3*, *BATF* and *ESRRA*. *IKZF3* lies in a known T1D susceptibility region, while *BATF* and *ESRRA* overlap other immune disease susceptibility regions, validating our approach and suggesting novel avenues of research for T1D.

**Availability and implementation**: VSEAMS is available for download (http://github.com/ollyburren/vseams).

**Contact**: chris.wallace@cimr.cam.ac.uk

**Supplementary information**: Supplementary data are available at *Bioinformatics* online.

## 1 INTRODUCTION

Genome-wide association studies (GWAS) have been successful in identifying loci associated with many phenotypes (Welter *et al.*, 2014), and summary statistics in the form of a list of single, single nucleotide polymorphism (SNP) *P*-values for each marker tested are increasingly becoming available in the public domain (Burren *et al.*, 2011; Okada *et al.*, 2014). In tandem with this, large amounts of functional genomic data across a wide variety of tissues and conditions are increasingly available through public repositories. Integrative methods that combine genome-wide genetic and genomic data have the potential to highlight functional genomic categories suitable for further study in relation to a given phenotype. This is particularly important in type 1 diabetes (T1D) where of the 49 susceptibility loci currently described (http://immunobase.org, accessed March 15, 2014), only 12 are consistent with a non-synonymous coding SNP as the causal variant. This is in accord with previous research (lari *et al.*, 2012; Schaub *et al.*, 2012), and indicates a central role for gene regulatory SNPs in the modulation of complex disease, where integrative methods have utility.

One such integrative approach is to modify gene set enrichment analyses methods (GSEA) developed for microarray pathway analysis (Subramanian *et al.*, 2005) for use with GWAS study datasets (Wang *et al.*, 2007). These approaches partner SNPs to genes based on public annotations and then test for differences in evidence of association between SNPs assigned to two sets of genes. There are several limitations with existing approaches. First, most methods require access to raw genotype data to correct for inter-SNP correlation due to linkage disequilibrium (LD). Raw genotype data are typically not available in the public domain, and this problem is compounded for meta-analysis–based GWAS, which combines multiple datasets. Second, the permutation-based approaches usually used to adjust for correlation are computationally expensive. Finally, classical gene set enrichment analysis is typically based on tests derived from the Kolmogorov–Smirnov, which is under powered. A need for simpler and more powerful methods has been identified (Irizarry *et al.*, 2009), but the proposed alternative, a *t*-test, has been criticized because it cannot cope with strong correlation between genes (Tamayo *et al.*, 2012).

We have previously used a Wilcoxon-based GSEA method to demonstrate enrichment for T1D association to a gene network driven by the transcription factor *IRF7* (Heinig *et al.*, 2010). The Wilcoxon test was used as a more powerful alternative to a Kolmogorov–Smirnov test, but the approach still required permutation to correct for the effects of LD. In this article, we describe an approximate method, that allows such tests to be performed with greater computational efficiency and, crucially, without access to raw genotype data, by extending an approach by Liu *et al.* (2010). We implement this extended approach in a freely available software pipeline VSEAMS. Although we have chosen the Wilcoxon test, the pipeline would be easily adaptable to any test of location such as a *t*-test.

Given previous evidence for the involvement of a network of genes linked to the transcription factor *IRF7* (Heinig *et al.*, 2010) in (T1D), we hypothesized that networks of genes dependent on other transcription factors might also show enrichment for T1D association. We used VSEAMS to test for enrichment of T1D association among the targets of 59 transcription factors identified through knock-down experiments in lymphoblastoid cell lines (Cusanovich *et al.*, 2014).

## 2 METHODS

### 2.1 Outline of existing Wilcoxon-based approach

Given two sets of genes (test and control), our task is to decide whether GWAS-association signals for a given trait differ between SNPs near test and control genes—a comparison of two distributions of *P*-values. We use a non-parametric test, the Wilcoxon rank sum test, to test a null hypothesis that these two distributions have equal medians, but any test of location could be used. The test statistic is denoted *W*. Its mean is known theoretically, but its variance is inflated when SNPs are in any degree of LD. To address this, Heinig *et al.* (2010) repeatedly permuted case/control status in a GWAS dataset to generate replicates of W under the null. A *Z*-score can be derived

(1) $$Z=\frac{\left(W-\mu_{0}\right)}{\sqrt{V}},$$

where *W* is the observed test statistic, *μ*_0_ is its theoretical mean, and *V* is its empirical variance derived from the replicates of *W*. V is problematic to compute because the univariate tests of association between SNPs are slow to compute and require access to raw the genotype data, which are not always available.

### 2.2 Approximation of *V* by V*

**Fig. 1.**
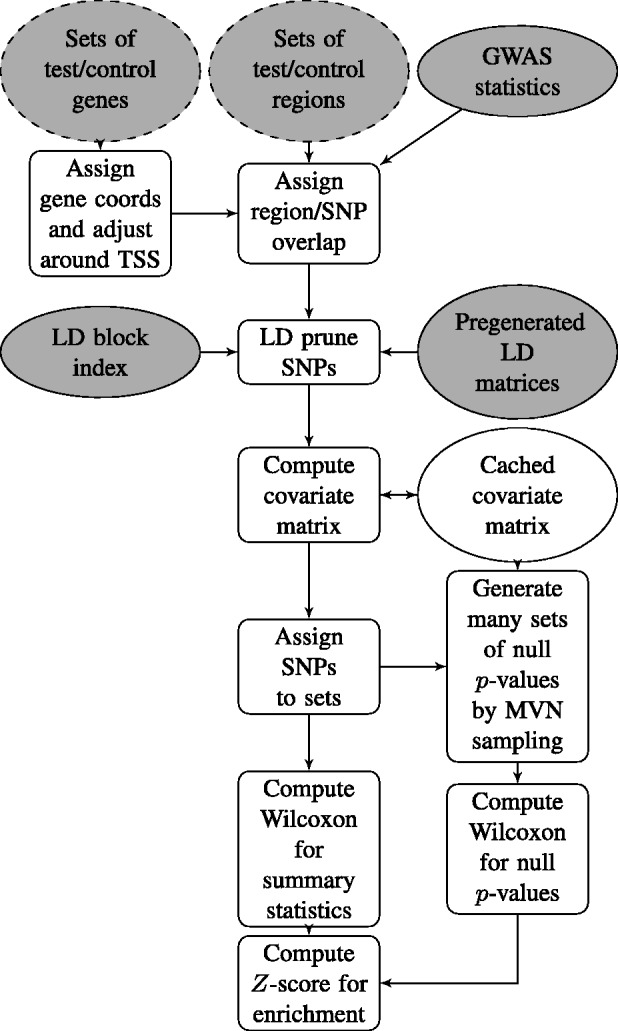
The VSEAMS pipeline; mandatory inputs are shaded grey; a dashed border indicates that one or the other input is required. VSEAMS takes as input either two lists of genes or two lists of regions for comparison. Given genes, regions are defined by taking gene coordinates ± 200 kb around the TSS. GWAS summary statistics (*P*-values) for SNPs in those regions are extracted. The observed Wilcoxon rank sum test statistic is compared with its null distribution determined by its theoretical mean and a variance derived by simulating null *P*-values with a correlation structure matching the underlying genotype structure. Caching of pregenerated LD matrices reduces computation time. A full description of each step is available in the Supplementary Information

VSEAMS removes the need to access the raw data by instead approximating *V* by V*. Given a matrix of pairwise genotype correlations at SNPs of interest, Σ, which may be derived from public data, we repeatedly sample $Z^{*}∼N(0,\Sigma )$, from which *P*-values can be derived in the usual manner. The link between correlation of genotypes and correlation of *Z*-scores is not entirely obvious and is derived in the supplementary information. These *P*-values can be combined in the same way as the observed data to give replicates of *W*, with V* equal to the empirical variance of these replicates. The full VSEAMS pipeline is described in Figure 1 and Supplementary Information.

### 2.3 Validation analyses

To validate the method, we used T1D GWAS data from the T1DGC study (see Supplementary Information) for which we have raw genotype data, ∼ 4000 cases and 4000 controls drawn from the UK population to compute and compare *V* and V* under different scenarios. SNP testing was conducted using the R package *snpStats*. To examine how VSEAMS performance is affected by gene set, we selected a random set of 200 protein coding genes (Supplementary Table S3) and generated 100 sets of 100 control and 100 test gene sets by randomly sampling from these 200 genes. For each set, we computed an enrichment *Z*-score using (i) VSEAMS and summary *P*-values and (ii) permuted case/control status and raw genotype data. To simulate modest enrichment, we repeated these analyses with the *P*-value for each SNP in the test set multiplied by 0.9.

To examine the effect of sample size and number of simulations, we created case/control genotype sets by randomly sampling a subset of cases and controls from the T1DGC dataset. For each sample size, we repeated this five times, and compared the Z statistics produced by VSEAMS (up to 10 000 simulations) or permutation methods (10 000 permutations).

### 2.4 Benchmark analyses

The VSEAMS pipeline is designed to run on a shared distributed computing platform, complicating runtime comparisons. We therefore designed a set of benchmarking tests to compare runtime for generating simulated and permuted test statistics under the null, the main methodological difference we wished to examine. We randomly selected 1000 LD blocks from the set of precomputed covariance matrices. Each underlying covariance matrix was filtered, so that only SNPs present and passing QC for the T1DGC study dataset were present. For each LD block, we created a set of corresponding genotype files using data from the T1DGC study.

In total, 14 753 SNPs were included over the 1000 randomly selected LD blocks. We examined the median runtime speed using the R package *microbenchmark* comparing the wgsea function pairtest() for the permutation method against the VSEAMS function mvs.perm() for 10 permutations or simulations, for a variable number of cases and controls. All benchmarks were run on a 4 Core AMD Opteron (2.8 GHz) with 32 GB of RAM. Each individual benchmark corresponds to the median time taken to generate 10 permutations or simulations for a given LD block for a given sample size. To estimate the total execution time for a given sample size, we summed median execution over all LD blocks.

### 2.5 Transcription factor gene set processing

Cusanovich *et al.* (2014) present the results of differential gene expression in siRNA knock-downs of 59 transcription factors and chromatin modifiers in lymphoblastoid cell lines. We downloaded results available in their Supplementary Table S3. For each transcription factor, we created a set of test genes that were differentially expressed at an false discovery rate (FDR) of 5%, making sure that the transcription factor itself was excluded from this list, using the *qvalues* R package. We created a control set by taking the remaining genes not in the test set and removing those with missing values or showing evidence of differential expression at an FDR of 10%. We ran each test/control set in parallel using VSEAMS, and extended gene regions to incorporate ±200 kb around gene transcriptional start site (TSS) to best capture regulatory variation (Stranger *et al.*, 2012). We simulated 100 000 replicates of *W* to confidently estimate V*.

## 3 RESULTS

### 3.1 VSEAMS pipeline

VSEAMS is a software pipeline implemented in R and Perl. To maximize performance, it uses grid-based computing and the *macd* queue submission manager. VSEAMS was developed to run using the Sun Grid Engine; however, *macd* is designed to be extensible to support other high-performance computing submission solutions. All software is available under open-source license (GPL v2) from (http://github.com/ollyburren/vseams and http://github.com/ollyburren/macd).

### 3.2 V* is a good approximation for *V* and computationally more efficient

**Fig. 2.**
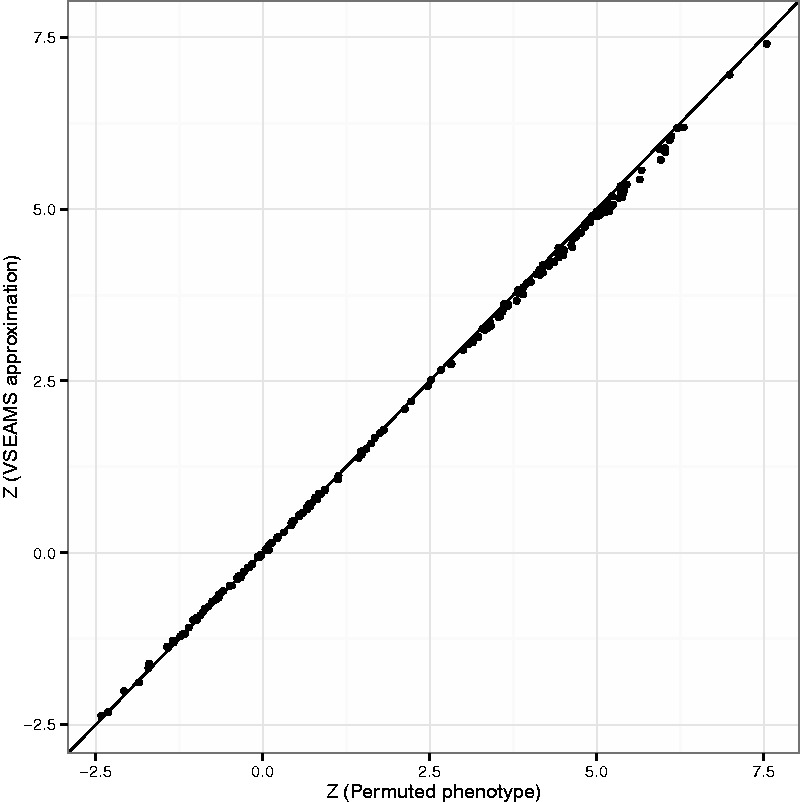
A comparison of *Z*-scores generated using permuted phenotype method (10 000 permutations) versus using summary *P*-values and VSEAMS (10 000 simulations) for T1DGC study, over 100 randomly generated gene sets

**Fig. 3.**
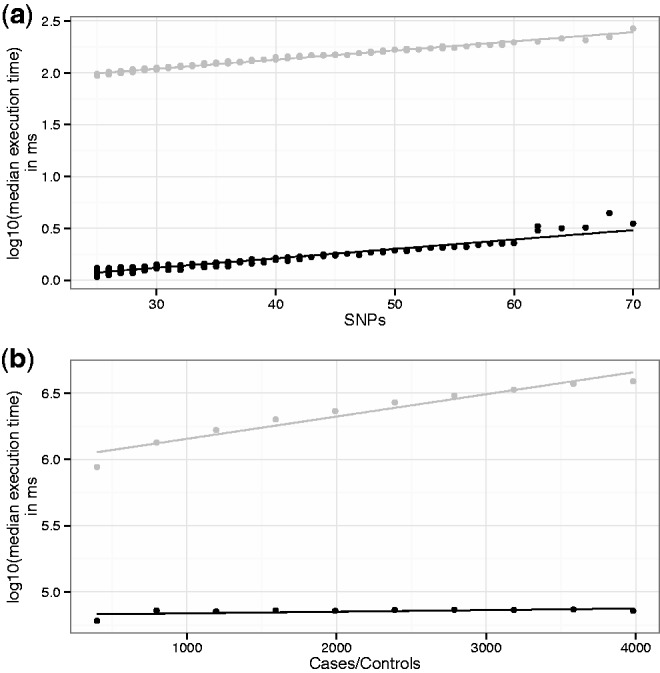
A runtime comparison of simulation using multivariate normal (black) versus permutation (grey) over 1000 randomly selected LD blocks. In both plots the *y*-axis is the median execution time over 10 iterations, and lines indicate the fitting of a linear model. Specifically, (**a**) details the effect of sample size on median execution time over 14 753 SNPs summed over all randomly selected LD blocks. (**b**) Shows the effect of SNP count on execution time for 4000 cases and controls for all 1000 randomly selected LD blocks

There is good correlation between results obtained from VSEAMS approximations and those from directly permuting genotype (Fig. 2). The simulation method implemented in VSEAMS is more efficient than a comparable permutation approach. Figure 3a shows that the generation of simulated statistics is faster than using permutation. Both methods exhibit a linear relationship with number of SNPs; however, the simulation is on average 100 times faster for a moderate GWAS of 4000 cases and 4000 controls (Fig. 3b). The permutation method runtime shows a linear relationship with sample size, whereas the simulation method runtime is independent of sample size, and is 10 times faster, even for 500 cases and controls.

### 3.3 T1D susceptibility enrichment in targets of the transcription factors *IKZF3*, *BATF* and *ESRRA*

**Fig. 4.**
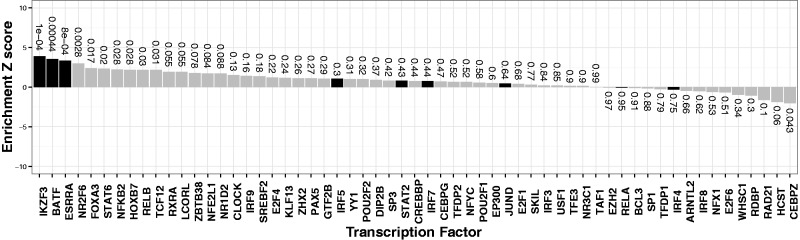
T1D susceptible SNP enrichment (excluding major histocompatibility complex (MHC)) within transcription factor perturbed gene sets from Cusanovich *et al.* (2014) SNPs are pruned on the basis of *r*^2^ threshold ≥0.95. A positive *Z*-score indicates enrichment, labels denote associated *P*-values. Black bars indicate that the knocked-down transcription factor overlaps a known autoimmune susceptibility locus curated in ImmunoBase

Genes perturbed by 3 of 59 transcription factors in knock-down experiments (Cusanovich *et al.*, 2014) were enriched for association with T1D (Fig. 4): *IKZF3* (*P* = 1.1 × $10^{-4},n=1798$), *BATF* (*P* = 4.4 × $10^{-4},n=210$) and *ESRRA* (*P* = 8.0 × $10^{-4},n=614$), where *n* is the number of genes in each set. Fourteen genes are common to all three sets (Supplementary Fig. S1 and Supplementary Table S2).

We used VSEAMS to prioritize individual genes within each significant set, selecting 95 genes of 2326 that exceeded Bonferroni threshold for that set (Supplementary Table S3). Of these, 63 overlap regions of known T1D susceptibility (http://immunobase.org accessed March15, 2014). We draw attention to 10 genes that have no conclusively established association to T1D but have been highlighted for other immune-modulated diseases in ImmunoBase (Table 1), three of which are implicated as candidate causal genes in one or more diseases: *TRAF3IP2* in psoriasis, ulcerative colitis and Crohn’s disease (Jostins *et al.*, 2012; Tsoi *et al.*, 2012), *ZNF438* in multiple sclerosis (IMSGC *et al.*, 2011) and *RUNX3* in ankylosing spondylitis and psoriasis (IGASC *et al.*, 2013; Tsoi *et al.*, 2012). The 22 remaining genes have no established association to autoimmune disease, their membership of functionally defined gene sets, which show overall association with T1D suggests that they are also worth noting.

**Table 1.** Genes with significant gene prioritization statistics identified from enriched gene sets not overlapping known T1D susceptibility loci

Transcription factor	Ensembl ID	HGNC symbol	*P* (empirical)	Coordinates	Disease overlap
*IKZF3*	ENSG00000056972	*TRAF3IP2*	$<10^{-6}$	chr6:111727481..112127481	CRO*^a^*, PSO*^a^*, UC*^a^*
*IKZF3*	ENSG00000183621	*ZNF438*	0.000008	chr10:31109136..31520866	MS*^a^*, RA
*IKZF3*	ENSG00000110344	*UBE4A*	$<10^{-6}$	chr11:118030300..118430300	CEL, MS, PBC, RA, SJO
*IKZF3*	ENSG00000108465	*CDK5RAP3*	0.000003	chr17:45845176..46245176	AS, MS
*IKZF3*	ENSG00000105655	*ISYNA1*	0.000006	chr19:18349111..18749111	MS
*IKZF3*	ENSG00000128268	*MGAT3*	0.000004	chr22:39653349..40053349	CD, PBC, UC
*BATF*	ENSG00000020633	*RUNX3*	0.000169	chr1:25091612..25491612	AS*^a^*, PS*^a^*
*BATF*	ENSG00000241685	*ARPC1A*	0.000218	chr7:98723521..99123521	CD, UC
*ESRRA*	ENSG00000213619	*NDUFS3*	0.000051	chr11:47386888..47786888*^b^*	MS
*ESRRA*	ENSG00000123444	*KBTBD4*	0.000082	chr11:47400567..47800567*^b^*	MS

*Note:* ‘Disease overlaps’ indicates that the interval defined overlaps a disease annotated in http://immunobase.org. Ankylosing spondylitis (AS), celiac disease (CEL), Crohn’s disease (CRO), juvenile idiopathic arthritis (JIA), multiple sclerosis (MS), psoriasis (PSO), rheumatoid arthritis (RA), ulcerative colitis (UC). Coordinates are given for build GRCh37. *^a^*Gene is implicated as causal in that disease. *^b^*Regions overlap.

### 3.4 Effect of sample size and simulation number

**Fig. 5.**
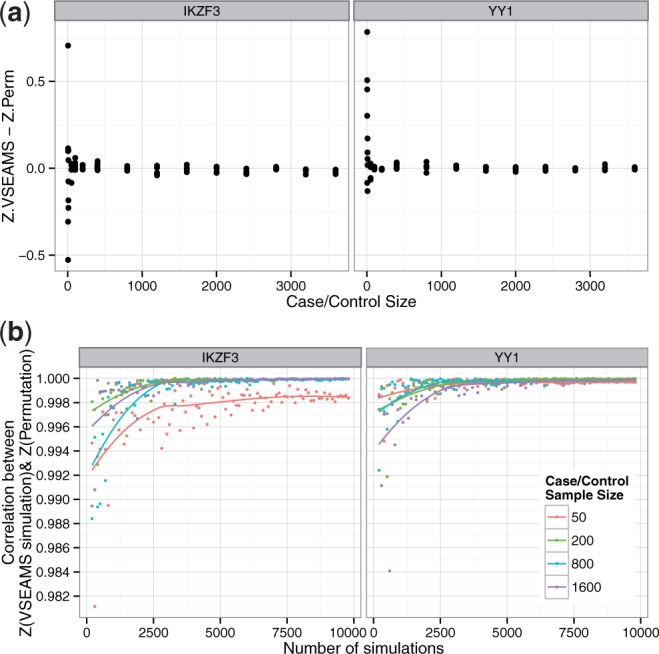
Comparison of VSEAMS and permuted phenotype methods with differing sample size, for example, gene sets, where enrichment is present (*IKZF3*) and absent (*YY1*). (**a**) Shows difference in *Z*-scores between both methods with 10 000 simulations and a variable sample size, with an equal number of cases and controls. (**b**) Shows how the correlation between *Z*-scores over a variable number of permutations varies with respect to sample size. The coloured lines represent a locally estimated scatterplot smoothing (LOESS) fitted model for each sample size

We picked two gene sets from the Cusanovich *et al.* (2014) dataset with similar test set SNP counts to examine the effect of sample size and gene set selection on VSEAMS performance, *IKZF3* as an example where enrichment is present and *YY1* where it is absent. Both sets exhibited similar behaviour. In general, we see that the number of permutations required for a stable correlation between permutation and VSEAMS *Z*-scores is independent of sample size and is mainly dependent on gene set, and for these gene sets, 5000 simulations seems sufficient to ensure VSEAMS is a good approximation for permutation. At sample sizes <10 with a fixed number of permutations, we observe a large difference between *Z*-scores generated using VSEAMS and permutation method (Fig. 5). Small sample sizes (<200) show reduced correlation even for large numbers of permutations.

## 4 DISCUSSION

Correlation is a problem for all enrichment analyses because it results in inflated test statistics compared with their theoretical distribution. This problem exists in GSEA of gene expression datasets, but is more pronounced for SNP data, in which historical recombination events produce LD patterns that are both complex and strong. The original GSEA method accounts for this correlation by permuting phenotypes and repeating the entire gene expression analysis multiple times (Subramanian *et al.*, 2005), an approach we also took in a previous variant set enrichment analysis (Heinig *et al.*, 2010). This computationally intensive approach seems required because permuting SNPs or genes directly destroys the correlation structure. Tests have been adapted for gene set enrichment that deal theoretically with the inflation of variance by estimating an average variance inflation factor (Wu and Smyth, 2012), but for SNPs, we do not believe a single variance inflation factor can capture the strength and highly variable correlation observed. Instead, in VSEAMS, we adapt a multivariate normal sampling approach, which we show is not only faster than phenotype permutation, but can be applied in the typical case where raw genotype data are not available. Our analyses indicate that the exact number of simulations required for a stable approximation of V* is specific to a gene set, but suggest that 5000 permutations is sufficient for the GWAS data we consider here. VSEAMS is designed not to require raw genotype data, and alternative methods to confirm sufficiency of simulation could be adopted from those developed in the Markov chain Monte Carlo (MCMC) literature (Geweke, 1992). Although this framework could equally be applied to parametric tests such as *t*-tests, we chose to concentrate on a non-parametric (Wilcoxon) test because it is more robust to occasional genotyping errors that may arise and that, without access to genotyping data, are impossible to check.

Although the selection of test sets is often straightforward, the selection of appropriate control sets tends not to be and requires careful understanding of the competitive hypothesis tested in enrichment studies and consideration of the appropriate control set. Here, we restricted our set of control genes to genes that were perturbed by at least one transcription factor in the lymphoblastoid cell line knock-down experiments (Cusanovich *et al.*, 2014). We encourage users to think carefully about the construction of control gene sets; for example, for microarray derived sets, we advocate matching on mean gene expression and coefficient of variation.

All three transcription factors we identify from Cusanovich *et al.* (2014) have been previously implicated in autoimmunity when cross-referenced with data from ImmunoBase (http://immunobase.org accessed April 3, 2014), providing validation of the method. *IKZF3* is a transcription factor located within a T1D susceptibility locus at 17q12 (Barrett *et al.*, 2009) and overlaps susceptibility loci for ulcerative colitis, Crohn’s disease, primary billiary cirrhosis and rheumatoid arthritis (Jostins *et al.*, 2012; Liu *et al.*, 2012; Stahl *et al.*, 2010). *IKZF3* is implicated in the regulation of B cell lymphocyte proliferation and differentiation (Morgan *et al.*, 1997). *BATF* overlaps rheumatoid arthritis and multiple sclerosis susceptibility loci at 14q24.3 (IMSGC *et al.*, 2011; Stahl *et al.*, 2010). Mice over expressing *Batf* show impaired T-cell development *in vitro* and no induction of IL-2 (Williams *et al.*, 2003). *ESRRA* overlaps alopecia areate, Crohn’s disease, multiple sclerosis and ulcerative colitis loci at 11q13.1 (IMSGC *et al.*, 2011; Jostins *et al.*, 2012; Petukhova *et al.*, 2010) and is a metabolic regulator of T-cell activation and differentiation (Michalek *et al.*, 2011). Future work will determine whether the enrichment pattern observed with T1D is shared with, or distinct from, other autoimmune traits.

The set of genes perturbed when *IRF7* is knocked down shows no evidence for enrichment, in contrast to our previous work (Heinig *et al.*, 2010). This likely reflects that the transcription factor experiments were performed in a lymphoblastoid cell line. The master regulator of the *IRF7* network previously described is *GPR183*, and is known to be activated by exposure to Epstein–Barr virus; therefore, *IRF7* responsiveness is likely to be altered in LCLs, which emphasizes a need for transcription factor function to be studied in primary cells.

Imprecise knowledge of regulatory variants for individual genes hampers any test of variant set enrichment. As regulatory variation may lie 200 kb from a gene (Stranger *et al.*, 2012), we use a large window to assign SNPs to genes. This increases the likelihood of overlapping regions occurring in test and control sets. We have implemented a random assignment strategy to mitigate this, and, although unbiased, this approach can result in a loss of power in the test for enrichment. Combination of chromatin state annotation with high-throughput chromatin conformation capture (‘Hi-C’) has the potential to allow better definition of genomic regions involved in regulating specific genes. This increased resolution will require a corresponding increase in GWAS resolution through the use of imputation. Additionally, as regulatory function varies in a cell-specific manner, annotation of multiple primary cell types and careful consideration of the biologically relevant cell types will be required. However, we expect this more precise definition of functional SNP sets will allow a sharp increase in the power of variant set enrichment analyses, and this will allow VSEAMS analyses to interpret functionally defined genetic regions by linking them to end-point phenotypes.

## Supplementary Material

Supplementary Data
