# Supplementary material for: VSEAMS: a pipeline for variant set enrichment analysis using summary GWAS data identifies IKZF3, BATF and ESRRA as key transcription factors in type 1 diabetes
Source: Bioinformatics. 2014 Aug 27;30(23):3342–8. doi: 10.1093/bioinformatics/btu571 (PMC4296156; doi:10.1093/bioinformatics/btu571)
Supplement: Supplementary Data [file supp_30_23_3342__index.html]

VSEAMS: A pipeline for variant set enrichment analysis using summary GWAS data identifies IKZF3, BATF and ESRRA as key transcription factors in type 1 diabetes — VSEAMS: a pipeline for variant set enrichment analysis using summary GWAS data identifies IKZF3, BATF and ESRRA as key transcription factors in type 1 diabetes — VSEAMS: a pipeline for variant set enrichment analysis using summary GWAS data identifies IKZF3, BATF and ESRRA as key transcription factors in type 1 diabetes — Supplementary Data 

# VSEAMS: a pipeline for variant set enrichment analysis using summary GWAS data identifies *IKZF3*, *BATF* and *ESRRA* as key transcription factors in type 1 diabetes

## Supplementary Data

files

**Files in this Data Supplement:**

- Supplementary Data - pdf file
