# Supplementary material for: VSEAMS: a pipeline for variant set enrichment analysis using summary GWAS data identifies IKZF3, BATF and ESRRA as key transcription factors in type 1 diabetes
Source: Bioinformatics. 2014 Aug 27;30(23):3342–8. doi: 10.1093/bioinformatics/btu571 (PMC4296156; doi:10.1093/bioinformatics/btu571)
Supplement: Supplementary Data [file supp_btu571_burren_et_al_vseams_supplementary_information.pdf]

Contents

1 Relationship of correlation of Z scores and SNP genotypes 1

2 VSEAMS pipeline 2

2.1 Creation of LD matrices and indices 2

2.2 LD pruning GWAS summary statistics 3

2.3 Creating SNP sets 3

2.4 Computing correlation matrices 3

2.5 Calculation of V^* 3

2.6 Enrichment Z-score 4

2.7 Prioritisation of genes within enriched gene sets 4

3 Type 1 diabetes GWAS datasets 4

4 Application of VSEAMS to meta-analysed GWAS 4

5 Supplementary Figures and Tables 5

1 Relationship of correlation of Z scores and SNP genotypes

We claim that the variance-covariance matrix for genetic association test statistics between a given phenotype and a number of SNPs may be approximated by V^*, the correlation matrix between genotypes at those SNPs. Here we set out a proof that the two are asymptotically equivalent by considering the standard Cochran-Armitage score test for association. Although this is just one of the tests used for genetic association, all single marker tests under the additive model are asymptotically equivalent.

Given a phenotype Y and genotype X, with observed values y_i and x_i measured on $i = 1, \ldots n$ individuals, the score statistic is

$$U_X = \sum_{i=1}^{n} y_i(x_i - \bar{x}) = \sum_{i=1}^{n} (y_i - \bar{y})(x_i - \bar{x})$$

and its variance is estimated by

$$V_X = (n-1)s_Y^2 s_X^2$$

where $s_X = \sum(x_i - \bar{x})^2$ and s_Y are asymptotically equal to $\mathrm{Var}\big(X\big)$ and $\mathrm{Var}\big(Y\big)$. Then

$$T_X = U_X/\sqrt{V_X} \sim N(0,1).$$

We define U_Z, V_Z, and T_Z similarly for another genotype variable, Z.

We want to show that $\mathrm{Cov}\,(T_X, T_Z)$ is asymptotically equal to the correlation between X and Z, $\frac{\mathrm{Cov}(X,Z)}{\sqrt{\mathrm{Var}\big(X\big)\,\mathrm{Var}\big(Z\big)}}$.

Let $V' = \sqrt{V_X V_Z}$. Then

$$
\begin{aligned}
\text{Cov}\,(T_X, T_Z) &= \frac{1}{V'}\,\text{Cov}(U_X, U_Z) \\
&= \frac{1}{V'}\,\text{Cov}\left(\sum_i (Y_i - \bar{Y})(X_i - \bar{X}),\ \sum (Y_i - \bar{Y})(Z_i - \bar{Z})\right) \\
&= \frac{1}{V'}\sum_{i,j}\text{Cov}\big((Y_i - \bar{Y})(X_i - \bar{X}),\ (Y_j - \bar{Y})(Z_j - \bar{Z})\big) \\
&= \frac{1}{V'}\sum_{i}\text{Cov}\big((Y_i - \bar{Y})(X_i - \bar{X}),\ (Y_i - \bar{Y})(Z_i - \bar{Z})\big) \\
&= \frac{1}{V'}\sum_{i} E((Y_i - \bar{Y})^2)\,\text{Cov}((X_i - \bar{X}),\ (Z_i - \bar{Z})) + E(X_i - \bar{X})E(Z_i - \bar{Z})\,\text{Var}\big(Y_i - \bar{Y}\big) \\
&= \frac{1}{V'}\sum_{i} E\left((Y_i - \bar{Y})^2\right)\ \sum_i \text{Cov}\left((X_i - \bar{X}),\ (Z_i - \bar{Z})\right)
\end{aligned}
$$

Now,

$$
\begin{aligned}
\text{Cov}((X_i - \bar{X}), (Z_i - \bar{Z})) &= \text{Cov}(X_i, Z_i) - \text{Cov}(X_i, \bar{Z}) - \text{Cov}(\bar{X}, Z_i) + \text{Cov}(\bar{X}, \bar{Z}) \\
&= \text{Cov}(X, Z) - \frac{1}{n}\text{Cov}(X, Z) - \frac{1}{n}\text{Cov}(X, Z) + \frac{1}{n}\text{Cov}(X, Z) \\
&= \frac{n-1}{n}\text{Cov}(X, Z)
\end{aligned}
$$

so, asymptotically,

$$
\begin{aligned}
\text{Cov}\,(T_X, T_Z) &= \frac{1}{(n-1)s_Y^2\sqrt{s_X^2 s_Z^2}}\frac{n(n-1)}{n}\text{Var}\big(Y\big)\,\text{Cov}(X, Z) \\
&= \frac{1}{\text{Var}\big(Y\big)\sqrt{V(X)V(Y)}}\text{Var}\big(Y\big)\,\text{Cov}(X, Z) \\
&= \frac{\text{Cov}(X, Z)}{\sqrt{\text{Var}\big(X\big)\,\text{Var}\big(Z\big)}}
\end{aligned}
$$

2 VSEAMS pipeline

2.1 Creation of LD matrices and indices

Previous software employing this multivariate normal simulation approach, VEGAS (Liu *et al.*, 2010), relies on a set of correlation matrices pregenerated on a predefined gene by gene basis using LD derived from HapMap phase 2 population data (HapMap, 2003). To make this approach more flexible to alteration in gene definition and applicable to SNPs not typed by the HapMap project, our method uses data from the 1000 Genomes Project (1000 Genomes Project Consortium *et al.*, 2010). Computing pairwise LD between all SNPs on a given chromosome is inefficient, therefore we split the genome into contiguous regions of length 0.1cM using HapMap recombination rate data. We assume there is no correlation between SNPs in different blocks. In practice, this assumption tends to be justified because most 0.1cM blocks are separated by recombination hot spots. We downloaded the EUR 1KG dataset in VCF form (http://www.sph.umich.edu/csg/abecasis/MACH/download/1000G.

2012-03-14.html accessed 02/01/2014) and computed pairwise LD (r^2) for each recombination region using tabix (Li, 2011) and Bioconductor R libraries (Gentleman *et al.*, 2004). Finally we created an index that allowed fast LD retrieval based on genomic coordinates. Using a 0.1 cM threshold, the human genome was split into 36,127 blocks, with a median of 507 SNPs in each block and and a median block length of 38.1Kb.

2.2 LD pruning GWAS summary statistics

As described above, LD between SNPs increases the variance of the test statistic. Some LD-based pruning of SNPs in the strongest LD can produce a more stable test statistic. VSEAMS achieves this by using the set of pregenerated LD matrices (see section 2.1) and hierarchical cluster analysis to select a set of tag SNPs at a user defined r^2 threshold. These are then taken forward for analysis. We recommend a relatively relaxed threshold of $r^2 >= 0.95$, removing just those SNPs in extremely strong LD.

2.3 Creating SNP sets

The first operation of VSEAMS takes a list of Ensembl gene (Flicek *et al.*, 2013) identifiers for both test and control sets and integrates these with bed-formatted GWAS data to provide a set of test and control SNPs. In order to capture potential regulatory sequences the software allows a user defined offset $\pm$ the transcriptional start site of each gene. Based on Stranger *et al.* (2012), which examines the overall distribution of the positions of regulatory SNPs and target genes, we recommend an offset of 200 kb. For even greater flexibility the software also accepts raw genomic coordinates to define test and control regions sets, and so is not limited to either a single source of annotation or even, gene-centric analysis. In some cases where genes or regions overlap, a variant is assigned to both test and control sets. To allow for this we randomly assign such SNPs to either test or control set. If such overlaps are substantial, we recommend repeating the analysis two or three times to check robustness of any result.

2.4 Computing correlation matrices

We employ a similar method to VEGAS (Liu *et al.*, 2010) to compute correlation matrices. Briefly, using the pregenerated index, we identify relevant pregenerated LD matrices (see section 2.1), and use Cholesky decomposition (implemented in the *corpcor* R package) to identify the nearest positive definite matrix. For efficiency these are computed once and stored as they are applicable for any future analysis using VSEAMS.

2.5 Calculation of V^*

The cached correlation matrices are then used to generate multivariate samples of correlated normal variables, Z, that mirror the LD-induced correlation in the observed data, using the *mvtnorm* R package (Genz and Bretz, 2009). These are converted to p values in the usual way and, using the R package *wgsea*, are used to compute replicates of W under the null. The empirical variance of these replicates is used to estimate V^*.

2.6 Enrichment Z-score

Using the empirical estimate of V^* above we derive a Z-score for enrichment.

$$Z = \frac{(W - \mu)}{\sqrt{V^*}}, \tag{1}$$

where W is the observed test statistic, μ is empirical mean and V^* is the estimated variance of the set of simulated Wilcoxon statistics. VSEAMS allows for analysis of multiple GWAS, for example, individual components of a meta analysis study, using van Elteren's method to calculate a combined Z-score (van Elteren, 1960), although we show that summary statistics from a meta analysis of multiple GWAS can be used directly.

2.7 Prioritisation of genes within enriched gene sets

If VSEAMS indicates enrichment of association in any functionally defined set of genomic regions or genes, we may wish to identify which members of that set are driving the enrichment. VSEAMS can be used to rank the genes based on summary statistics. For each gene/region in the enriched test set VSEAMS computes $\bar{P} = \text{mean}(-\log(p))$ over SNPs assigned to that gene, and using simulations already available we compute n sets of $\bar{P}_i^* = \text{mean}(-\log(p_i^*))$. An empirical p-value is given by

$$\frac{1}{n}\sum_{i=1}^{n} I(\bar{P}_i^* > \bar{P})$$

where i indexes the n simulated datasets and $I()$ is an indicator function. Note that as $p \sim U[0, 1]$ under a null of no association, $-\log(p) \sim Exp(1)$, and so $\bar{P}$ is expected to be close to 1 where a gene is not associated with a given trait.

3 Type 1 diabetes GWAS datasets

Barrett *et al.* (2009) published a meta analysis of three T1D GWAS, comprising one study using the Affymetrix 500k, (Wellcome Trust Case Control Consortium, 2007, WTCCC), and two which used the Illumina 550k chip. One of these selected cases from Genetics of Kidneys in Diabetes (GoKinD) study of diabetic nephropathy and reference samples from the National Institute of Mental Health (NIMH) study and the other used samples from the Type 1 Diabetes Genetic Consortium (Cooper *et al.*, 2008, T1DGC). Genotypes were imputed to allow all SNPs genotyped in either study to be meta analysed in a total of 7,514 cases and 9,045 controls. Due to both its large effect on T1D risk and the extended LD across the MHC region, we excluded all SNPs in a window chr6:25Mb..35Mb (GRCh37) from analysis. We downloaded GWAS summary statistics from T1DBase.org (Burren *et al.*, 2011) and applied quality control thresholds as described in Barrett *et al.* (2009).

4 Application of VSEAMS to meta-analysed GWAS

GWAS meta-analysis often contains imputed datasets where concerns about different genotyping chips may arise. To confirm that VSEAMS is applicable such a meta-analysis, we generated a set of 1000 control/test gene sets by randomly sampling the set of 200 genes detailed in supplementary table 3. We then computed enrichment p values for each set using the Barrett *et al.* (2009) meta analysis p

values using 100 simulations of W to estimate V^*. Supplementary figure 2 shows that for the dataset considered such an approximation is appropriate.

References

1000 Genomes Project Consortium *et al.* (2010). A map of human genome variation from population-scale sequencing. *Nature*, **467**, 1061–73.

Barrett, J.C. *et al.* (2009). Genome-wide association study and meta-analysis find that over 40 loci affect risk of type 1 diabetes. *Nat. Genet.*, **41**, 703–7.

Burren, O.S. *et al.* (2011). T1DBase: update 2011, organization and presentation of large-scale data sets for type 1 diabetes research. *Nucleic Acids Res.*, **39**, D997–1001.

Cooper, J.D. *et al.* (2008). Meta-analysis of genome-wide association study data identifies additional type 1 diabetes risk loci. *Nat. Genet.*, **40**, 1399–401.

Flicek, P. *et al.* (2013). Ensembl 2013. *Nucleic Acids Res*, **41**, D48–D55.

Gentleman, R.C. *et al.* (2004). Bioconductor: open software development for computational biology and bioinformatics. *Genome Biol*, **5**, R80.

Genz, A. and Bretz, F. (2009). *Computation of Multivariate Normal and t Probabilities*. Lecture Notes in Statistics. Springer-Verlag, Heidelberg. ISBN 978-3-642-01688-2.

HapMap (2003). The International HapMap Project. *Nature*, **426**, 789–96.

Li, H. (2011). Tabix: fast retrieval of sequence features from generic TAB-delimited files. *Bioinformatics*, **27**, 718–9.

Liu, J.Z. *et al.* (2010). A versatile gene-based test for genome-wide association studies. *Am. J. Hum. Genet.*, **87**, 139–45.

Stranger, B.E. *et al.* (2012). Patterns of cis regulatory variation in diverse human populations. *PLoS Genet.*, **8**, e1002639.

van Elteren, P. (1960). On the combination of independent two sample tests of Wilcoxon. *Bulletin of the International Statistical Institute*, **37**, 351–361.

Wellcome Trust Case Control Consortium (2007). Genome-wide association study of 14,000 cases of seven common diseases and 3,000 shared controls. *Nature*, **447**, 661–78.

5 Supplementary Figures and Tables

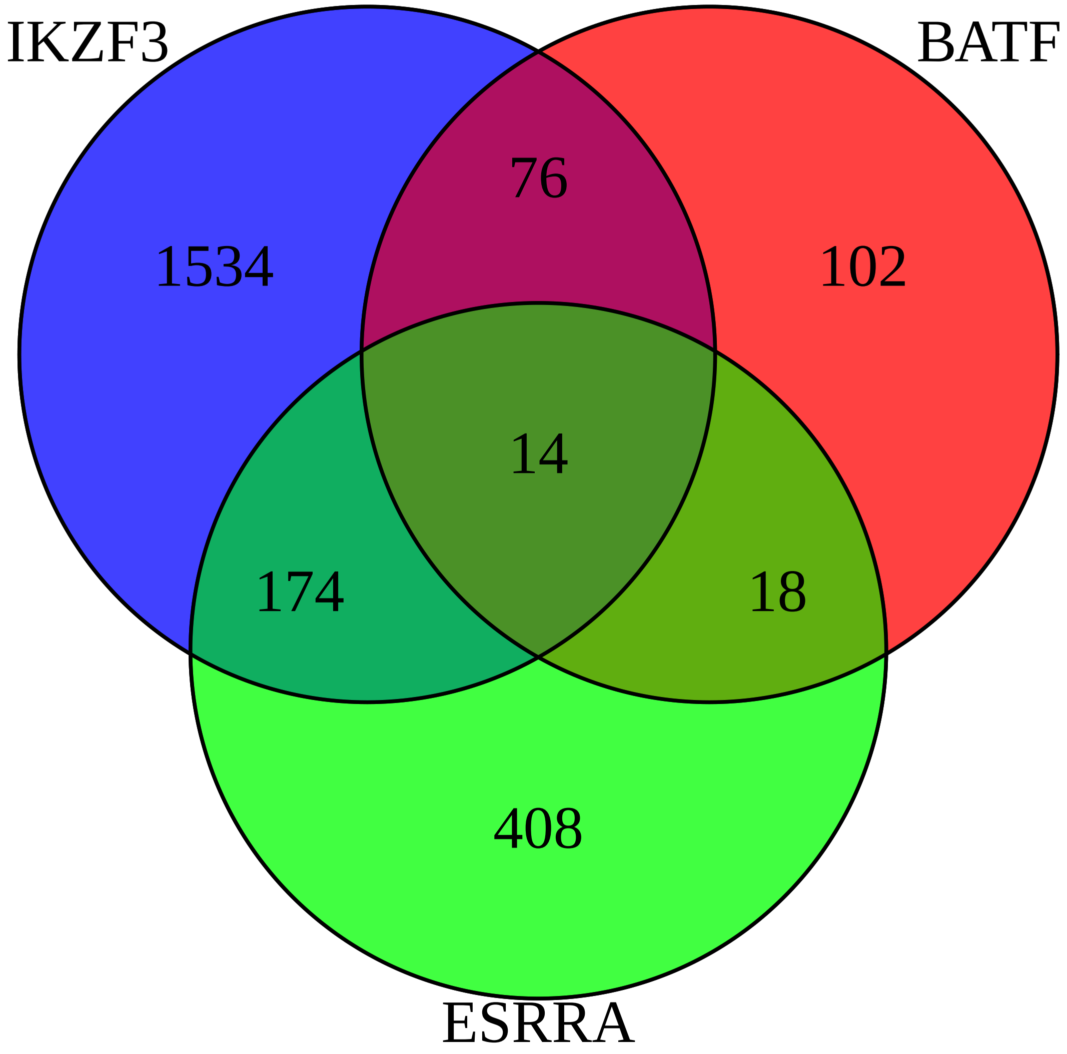

Supplementary Figure 1: Overlap between the different gene sets showing significant enrichment for type 1 diabetes association from *Cusanovich et al.*, *IKZF3*(n=1798), *BATF*(n=210) and *ESRAA*(n=614).

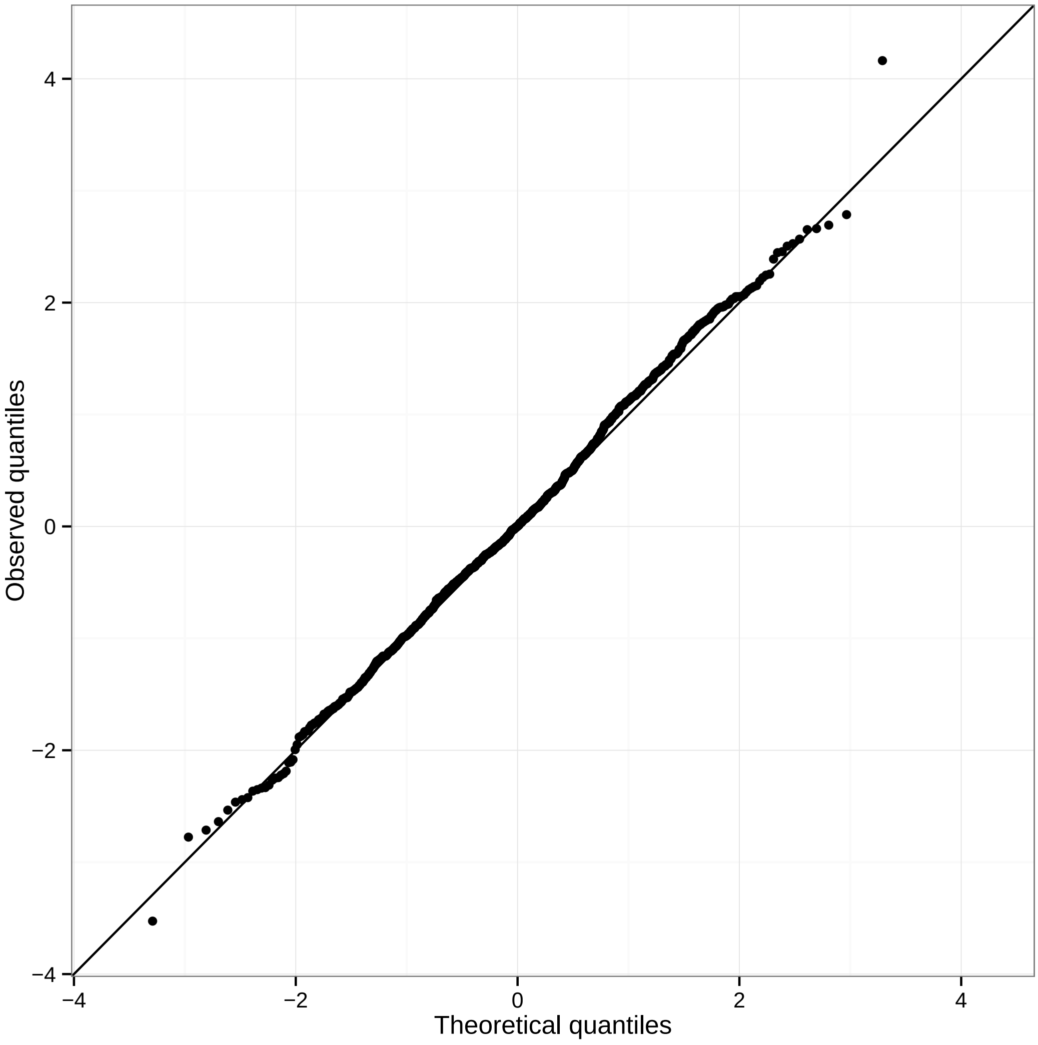

Supplementary Figure 2: A qq plot using VSEAMS (100 permutations) applied to the meta analysis of Barrett *et al.* (2009) for 1000 gene sets.

Number	Chromosome	Start	End	Strand	Ensembl ID	HGNC ID
1	chr1	8412457	9077702	-	ENSG00000142599	RERE
2	chr1	10290159	10690159	+	ENSG00000175279	APITD1
3	chr1	13190765	13590765	-	ENSG00000182330	PRAMEF8
4	chr1	15711605	16111605	-	ENSG00000116771	AGMAT
5	chr1	25464408	25864408	+	ENSG00000183726	TMEM50A
6	chr1	32487529	32887529	+	ENSG00000084623	EIF3I
7	chr1	32617122	33017122	+	ENSG00000162526	TSSK3
8	chr1	33346597	33746597	-	ENSG00000004455	AK2
9	chr1	40742887	41142887	+	ENSG00000187815	ZFP69
10	chr1	92214928	92614928	+	ENSG00000137948	BRDT
11	chr1	93445476	93845476	+	ENSG00000122483	CCDC18
12	chr1	109958726	110358726	+	ENSG00000116337	AMPD2
13	chr1	110750564	111150564	-	ENSG00000134248	LAMTOR5
14	chr1	112809163	113209163	+	ENSG00000134245	WNT2B
15	chr1	114853781	115253781	-	ENSG00000197323	TRIM33
16	chr1	145089772	145489772	+	ENSG00000163386	NBPF10
17	chr1	151145209	151545209	-	ENSG00000143416	SELENBP1
18	chr1	153431130	153831130	+	ENSG00000143553	SNAPIN
19	chr1	153695451	154095451	-	ENSG00000143614	GATAD2B
20	chr1	157322310	157722310	-	ENSG00000143297	FCRL5
21	chr1	178850512	179250512	+	ENSG00000186283	TOR3A
22	chr1	206943970	207343970	-	ENSG00000162897	FCAMR
23	chr1	211231719	211631719	+	ENSG00000117625	RCOR3
24	chr1	212258879	212658879	+	ENSG00000066027	PPP2R5A
25	chr1	223366715	223766715	+	ENSG00000178395	C1orf65
26	chr2	105682585	106082585	-	ENSG00000268809	
27	chr2	113322254	113722254	-	ENSG00000169607	CKAP2L
28	chr2	127976003	128376003	+	ENSG00000115718	PROC
29	chr2	130739310	131139310	+	ENSG00000152082	MZT2B
30	chr2	171847333	172287824	-	ENSG00000198586	TLK1
31	chr2	219997899	220397899	-	ENSG00000182698	RESP18
32	chr3	32233163	32633163	+	ENSG00000153551	CMTM7
33	chr3	42747223	43147223	+	ENSG00000182983	ZNF662
34	chr3	45727996	46127996	+	ENSG00000173585	CCR9
35	chr3	49767606	50167606	-	ENSG00000164077	MON1A
36	chr3	111497857	111897857	+	ENSG00000144827	ABHD10
37	chr3	124798021	125198021	-	ENSG00000221955	SLC12A8
38	chr3	125039041	125439041	-	ENSG00000114520	SNX4
39	chr3	125455882	125855882	-	ENSG00000189366	ALG1L
40	chr3	133571028	133971028	-	ENSG00000174640	SLCO2A1
41	chr3	145768966	146168966	-	ENSG00000114698	PLSCR4
42	chr3	148383043	148783043	+	ENSG00000163751	CPA3
43	chr3	183779251	184179251	-	ENSG00000163888	CAMK2N2
44	chr3	189840264	190240264	-	ENSG00000163347	CLDN1
45	chr4	6517842	6917842	+	ENSG00000186222	BLOC1S4
46	chr4	9145874	9545874	+	ENSG00000235780	USP17L27
47	chr4	38984024	39384024	+	ENSG00000157796	WDR19

Continued on next page

Supplementary Table 1 – continued from previous page

Number	Chromosome	Start	End	Strand	Ensembl ID	HGNC ID
48	chr4	44175926	44650824	-	ENSG00000183783	KCTD8
49	chr4	71568043	71968043	+	ENSG00000173542	MOB1B
50	chr4	140022609	140422609	+	ENSG00000164134	NAA15
51	chr5	133140824	133540824	-	ENSG00000213585	VDAC1
52	chr5	137678989	138078989	-	ENSG00000120705	ETF1
53	chr5	139287362	139687362	+	ENSG00000185129	PURA
54	chr5	140007563	140407563	+	ENSG00000081842	PCDHA6
55	chr5	176126333	176526333	-	ENSG00000160883	HK3
56	chr5	176360026	176760026	+	ENSG00000165671	NSD1
57	chr5	176578853	176978853	-	ENSG00000169223	LMAN2
58	chr5	177357997	177757997	+	ENSG00000145916	RMND5B
59	chr6	4506393	4955785	+	ENSG00000153046	CDYL
60	chr6	25833796	26233796	-	ENSG00000137259	HIST1H2AB
61	chr6	29490552	29890552	+	ENSG00000204642	HLA-F
62	chr6	32843703	33243703	+	ENSG00000223865	HLA-DPB1
63	chr6	79587953	79987953	-	ENSG00000146247	PHIP
64	chr6	91096764	91496764	-	ENSG00000135341	MAP3K7
65	chr6	111103218	111503218	+	ENSG00000197498	RPF2
66	chr6	116666773	117066773	-	ENSG00000173626	TRAPPC3L
67	chr6	130134844	130534844	+	ENSG00000198945	L3MBTL3
68	chr6	131256806	131656806	+	ENSG00000118507	AKAP7
69	chr6	132884598	133284598	-	ENSG00000112303	VNN2
70	chr6	139413276	139813276	-	ENSG00000164440	TXLNB
71	chr6	142209936	142609936	-	ENSG00000135577	NMBR
72	chr6	153352455	153752455	+	ENSG00000213121	
73	chr6	165523096	165923096	-	ENSG00000112539	C6orf118
74	chr7	5265045	5665045	-	ENSG00000182095	TNRC18
75	chr7	6188612	6588612	-	ENSG00000178397	FAM220A
76	chr7	21382833	21941457	+	ENSG00000105877	DNAH11
77	chr7	30123923	30523923	+	ENSG00000180233	ZNRF2
78	chr7	32733743	33133743	-	ENSG00000170852	KBTBD2
79	chr7	35535181	35935181	-	ENSG00000122557	HERPUD2
80	chr7	36893961	37688852	-	ENSG00000155849	ELMO1
81	chr7	44380914	44780914	-	ENSG00000015520	NPC1L1
82	chr7	44421886	44821886	-	ENSG00000158604	TMED4
83	chr7	47928225	48328225	+	ENSG00000183696	UPP1
84	chr7	72897783	73297783	-	ENSG00000176410	DNAJC30
85	chr7	89813035	90213035	+	ENSG00000157224	CLDN12
86	chr7	107184142	107584142	+	ENSG00000105879	CBLL1
87	chr7	107243670	107643670	-	ENSG00000091138	SLC26A3
88	chr7	108010110	108410110	-	ENSG00000135241	PNPLA8
89	chr7	127801739	128201739	-	ENSG00000224940	PRRT4
90	chr7	140190577	140590577	+	ENSG00000090266	NDUFB2
91	chr8	6595860	6995860	-	ENSG00000164821	DEFA4
92	chr8	6637602	7037602	-	ENSG00000206047	DEFA1
93	chr8	22235792	22635792	+	ENSG00000120913	PDLIM2

Continued on next page

Supplementary Table 1 – continued from previous page

Number	Chromosome	Start	End	Strand	Ensembl ID	HGNC ID
94	chr8	63881112	64281112	+	ENSG00000185728	YTHDF3
95	chr8	94567072	94967072	+	ENSG00000164953	TMEM67
96	chr8	110146614	110546614	-	ENSG00000120526	NUDCD1
97	chr8	110788076	111188076	-	ENSG00000164794	KCNV1
98	chr8	117687105	118087105	-	ENSG00000164754	RAD21
99	chr8	133572958	133972958	-	ENSG00000165071	TMEM71
100	chr8	141445718	141845718	-	ENSG00000123908	AGO2
101	chr9	15311017	15711017	-	ENSG00000164985	PSIP1
102	chr9	19030433	19430433	+	ENSG00000137145	DENND4C
103	chr9	46189110	46589110	+	ENSG00000231997	FAM27D1
104	chr9	114942217	115342217	+	ENSG00000119471	HSDL2
105	chr9	130287152	130687152	-	ENSG00000187024	PTRH1
106	chr9	138170925	138570925	+	ENSG00000196422	PPP1R26
107	chr9	138331386	138731386	-	ENSG00000204007	GLT6D1
108	chr9	139068133	139468133	-	ENSG00000187796	CARD9
109	chr9	139765040	140165040	-	ENSG00000186193	SAPCD2
110	chr10	37947034	38347034	-	ENSG00000198105	ZNF248
111	chr10	70739988	71139988	+	ENSG00000156502	SUPV3L1
112	chr10	73411126	73811126	-	ENSG00000197746	PSAP
113	chr10	74812451	75212451	-	ENSG00000182180	MRPS16
114	chr10	112431565	112831565	+	ENSG00000150593	PDCD4
115	chr11	724894	1124894	+	ENSG00000183020	AP2A2
116	chr11	5928914	6328914	+	ENSG00000180919	OR56B4
117	chr11	31331297	31805546	+	ENSG00000109911	ELP4
118	chr11	47387121	47787121	-	ENSG00000149187	CELF1
119	chr11	48146472	48546472	+	ENSG00000176547	OR4C3
120	chr11	55705194	56105194	-	ENSG00000167822	OR8J3
121	chr11	72185635	72585635	-	ENSG00000186642	PDE2A
122	chr11	89243467	89643467	+	ENSG00000214414	TRIM77
123	chr11	95709762	96276344	-	ENSG00000184384	MAML2
124	chr11	111549659	111949659	+	ENSG00000137720	C11orf1
125	chr11	111757497	112157497	+	ENSG00000204370	SDHD
126	chr11	128575930	128975930	-	ENSG00000174370	C11orf45
127	chr12	899219	1299219	-	ENSG00000002016	RAD52
128	chr12	4558213	4958213	-	ENSG00000111254	AKAP3
129	chr12	10124737	10524737	-	ENSG00000173391	OLR1
130	chr12	21454603	21854603	-	ENSG00000004700	RECQL
131	chr12	55620038	56020038	+	ENSG00000185821	OR6C76
132	chr12	56839798	57239798	-	ENSG00000110955	ATP5B
133	chr12	89547048	89947048	-	ENSG00000139318	DUSP6
134	chr12	105180088	105580088	+	ENSG00000151131	C12orf45
135	chr12	105429068	105829068	+	ENSG00000235162	C12orf75
136	chr12	120439038	120839038	-	ENSG00000089157	RPLP0
137	chr12	123001439	123401439	-	ENSG00000255398	HCAR3
138	chr13	31280328	31680328	+	ENSG00000102802	MEDAG
139	chr13	41506882	41906882	-	ENSG00000165572	KBTBD6

Continued on next page

Supplementary Table 1 – continued from previous page

Number	Chromosome	Start	End	Strand	Ensembl ID	HGNC ID
140	chr13	49622047	50022047	+	ENSG00000102543	CDADC1
141	chr13	52178293	52578293	-	ENSG00000102796	DHRS12
142	chr13	53026844	53426844	+	ENSG00000165416	SUGT1
143	chr13	77260540	77660540	-	ENSG00000178695	KCTD12
144	chr14	24700160	25100160	-	ENSG00000139899	CBLN3
145	chr14	77382911	77782911	+	ENSG00000165548	TMEM63C
146	chr14	94396590	94796590	-	ENSG00000119632	IFI27L2
147	chr14	102858998	103258998	+	ENSG00000089902	RCOR1
148	chr14	104846021	105246021	+	ENSG00000184601	C14orf180
149	chr15	27016429	27778373	+	ENSG00000182256	GABRG3
150	chr15	28929629	29410518	+	ENSG00000034053	APBA2
151	chr15	40026347	40426347	+	ENSG00000128829	EIF2AK4
152	chr15	40374787	40774787	-	ENSG00000230778	ANKRD63
153	chr15	59239899	59639899	+	ENSG00000268327	
154	chr15	89564982	89964982	-	ENSG00000140522	RLBP1
155	chr16	-72994	327006	+	ENSG00000103152	MPG
156	chr16	237113	637113	-	ENSG00000129925	TMEM8A
157	chr16	21818959	22218959	+	ENSG00000185716	C16orf52
158	chr16	29266285	29666285	-	ENSG00000183336	BOLA2
159	chr16	30917428	31317428	+	ENSG00000103507	BCKDK
160	chr16	81572702	81991899	+	ENSG00000197943	PLCG2
161	chr17	2296504	2696504	+	ENSG00000007168	PAFAH1B1
162	chr17	6920444	7320444	+	ENSG00000072778	ACADVL
163	chr17	7893564	8293564	-	ENSG00000196544	C17orf59
164	chr17	8080029	8480029	-	ENSG00000184619	KRBA2
165	chr17	18180051	18580051	+	ENSG00000171916	LGALS9C
166	chr17	18653658	19053658	+	ENSG00000154025	SLC5A10
167	chr17	30269473	30669473	+	ENSG00000126858	RHOT1
168	chr17	33500720	33900720	-	ENSG00000172716	SLFN11
169	chr17	39259103	39659103	-	ENSG00000212658	KRTAP29-1
170	chr17	46408359	46808359	-	ENSG00000120094	HOXB1
171	chr17	48512138	48912138	+	ENSG00000108846	ABCC3
172	chr17	52846088	53246088	+	ENSG00000166263	STXBP4
173	chr17	61759295	62159295	-	ENSG00000136487	GH2
174	chr17	72158085	72558085	-	ENSG00000204347	BTBD17
175	chr17	77085427	77813550	-	ENSG00000167281	RBFOX3
176	chr17	79415495	79815495	-	ENSG00000182446	NPLOC4
177	chr18	12502776	12902776	-	ENSG00000101624	CEP76
178	chr19	5140814	5540814	-	ENSG00000105426	PTPRS
179	chr19	8254865	8654865	+	ENSG00000185236	RAB11B
180	chr19	8278154	8678154	+	ENSG00000099785	MARCH2
181	chr19	11346109	11746109	+	ENSG00000130175	PRKCSH
182	chr19	40524306	40924306	-	ENSG00000174521	TTC9B
183	chr19	45481495	45881495	-	ENSG00000007255	TRAPPC6A
184	chr19	49175649	49575649	+	ENSG00000087074	PPP1R15A
185	chr20	32199110	32599110	+	ENSG00000101421	CHMP4B

Continued on next page

Supplementary Table 1 – continued from previous page

Number	Chromosome	Start	End	Strand	Ensembl ID	HGNC ID
186	chr20	55995632	56395632	-	ENSG00000124256	ZBP1
187	chr20	60595323	60995323	-	ENSG00000101180	HRH3
188	chr20	60613580	61013580	+	ENSG00000130703	OSBPL2
189	chr21	42533870	42933870	+	ENSG00000183486	MX2
190	chr21	47152477	47552477	-	ENSG00000268040	
191	chr22	17365844	17765844	+	ENSG00000177663	IL17RA
192	chr22	23893279	24293279	-	ENSG00000187792	ZNF70
193	chr22	41817100	42217100	-	ENSG00000100418	DESI1
194	chrX	69279654	69679654	-	ENSG00000186912	P2RY4
195	chrX	71549366	71992953	-	ENSG00000147099	HDAC8
196	chrX	99984422	100384422	-	ENSG00000182489	XKRX
197	chrX	105845910	106245910	+	ENSG00000133138	TBC1D8B
198	chrX	114054540	114454540	-	ENSG00000123496	IL13RA2
199	chrX	118084542	118484542	-	ENSG00000250423	KIAA1210
200	chrX	152754465	153154465	-	ENSG00000130822	PNCK

Supplementary Table 1: List of 200 gene regions used for simulation. Positions are given with respect to GRCh37, Ensembl ID's refer to release 75 of Ensembl

Ensembl ID	HGNC Symbol	Strand	Coordinates	Disease Overlap
ENSG00000116574	RHOU	+	chr1:228870824..228882416	
ENSG00000121957	GPSM2	+	chr1:109417972..109477167	
ENSG00000188404	SELL	-	chr1:169659808..169680839	
ENSG00000151694	ADAM17	-	chr2:9628615..9695921	
ENSG00000085719	CPNE3	+	chr8:87497059..87573726	
ENSG00000136982	DSCC1	-	chr8:120846216..120868250	
ENSG00000107223	EDF1	-	chr9:139756571..139760738	
ENSG00000187742	SECISBP2	+	chr9:91933421..91974557	
ENSG00000149289	ZC3H12C	+	chr11:109964087..110042566	PSO
ENSG00000181019	NQO1	-	chr16:69740899..69760854	
ENSG00000198417	MT1F	+	chr16:56691606..56694610	
ENSG00000224161	RPS26P54	+	chr18:57428790..57429137	
ENSG00000160190	SLC37A1	+	chr21:43916118..44001550	
ENSG00000102393	GLA	-	chrX:100652791..100662913	

Supplementary Table 2: 14 Genes overlapping all 3 transcription factor knockdown gene sets enriched for T1D associated variants, Psoriasis (PSO)

Transcription Factor	Ensembl ID	Name	$P_{.empirical}$	Coordinates	Disease Overlap	Band
IKZF3	ENSG00000116560	SFPQ	7e-06	chr1:35458749..35858749		1p34.3
IKZF3	ENSG00000020129	NCDN	1e-06	chr1:35823074..36223074		1p34.3
IKZF3	ENSG00000126067	PSMB2	$> 10^{-6}$	chr1:35907445..36307445		1p34.3
IKZF3	ENSG00000236887		$> 10^{-6}$	chr1:113541447..113941447	ATD, CRO, JIA, RA, SLE, T1D	1p13.2
IKZF3	ENSG00000116793	PHTF1	$> 10^{-6}$	chr1:114102111..114502111	ATD, CRO, JIA, RA, SLE, T1D	1p13.2
IKZF3	ENSG00000134242	PTPN22	$> 10^{-6}$	chr1:114214381..114614381	ATD, CRO, JIA, RA, SLE, T1D, VIT	1p13.2
IKZF3	ENSG00000143321	HDGF	$> 10^{-6}$	chr1:156536717..156936717		1q23.1
IKZF3	ENSG00000162889	MAPKAPK2	3e-06	chr1:206658289..207058289	CRO, SLE, T1D, UC	1q32.1
IKZF3	ENSG00000123685	BATF3	2.5e-05	chr1:212673327..213073327		1q32.3
IKZF3	ENSG00000138031	ADCY3	2.5e-05	chr2:24942708..25342708	CRO, MS, T1D, UC	2p23.3
IKZF3	ENSG00000115137	DNAJC27	1.5e-05	chr2:24994963..25394963	CRO, MS, T1D, UC	2p23.3
IKZF3	ENSG00000121966	CXCR4	1e-06	chr2:136675735..137075735		2q21.3
IKZF3	ENSG00000163600	ICOS	$> 10^{-6}$	chr2:204601471..205001471	AA, ATD, CEL, PSC, RA, T1D	2q33.2
IKZF3	ENSG00000121807	CCR2	$> 10^{-6}$	chr3:46195225..46595225	CEL, JIA, T1D	3p21.31
IKZF3	ENSG00000181722	ZBTB20	$> 10^{-6}$	chr3:114056941..115066118		3q13.31
IKZF3	ENSG00000056972	TRAF3IP2	$> 10^{-6}$	chr6:111727481..112127481	CRO, PSO, UC	6q21
IKZF3	ENSG00000146433	TMEM181	$> 10^{-6}$	chr6:158757468..159157468		6q25.3
IKZF3	ENSG00000185811	IKZF1	$> 10^{-6}$	chr7:50143720..50543720	CRO, MS, T1D	7p12.2
IKZF3	ENSG00000197157	SND1	1e-06	chr7:127092234..127732661		7q31.33
IKZF3	ENSG00000164733	CTSB	$> 10^{-6}$	chr8:11526957..11926957		8p23.1
IKZF3	ENSG00000107249	GLIS3	$> 10^{-6}$	chr9:3824127..4548392	T1D	9p24.2
IKZF3	ENSG00000165006	UBAP1	2e-05	chr9:33979003..34379003		9p13.3
IKZF3	ENSG00000134453	RBM17	$> 10^{-6}$	chr10:5930950..6330950	AA, ATD, CRO, JIA, MS, PSC, RA, T1D	10p15.1
IKZF3	ENSG00000170525	PFKFB3	$> 10^{-6}$	chr10:5986881..6386881	AA, ATD, CRO, JIA, MS, PSC, RA, T1D	10p15.1
IKZF3	ENSG00000065675	PRKCQ	$> 10^{-6}$	chr10:6422263..6822263	CEL, T1D	10p15.1
IKZF3	ENSG00000183621	ZNF438	8e-06	chr10:31109136..31520866	MS, RA	10p11.23
IKZF3	ENSG00000160584	SIK3	1.9e-05	chr11:116714118..117169153		11q23.3
IKZF3	ENSG00000110344	UBE4A	$> 10^{-6}$	chr11:118030300..118430300	CEL, MS, PBC, RA, SJO	11q23.3
IKZF3	ENSG00000184293	CLECL1	$> 10^{-6}$	chr12:9685895..10085895	MS, T1D	12p13.31
IKZF3	ENSG00000139626	ITGB7	6e-06	chr12:53401091..53801091		12q13.13
IKZF3	ENSG00000185664	PMEL	$> 10^{-6}$	chr12:56167101..56567101	AA, PSO, T1D	12q13.2

Continued on next page

Supplementary Table 3 – continued from previous page

Transcription Factor	Ensembl ID	Name	$P_{.empirical}$	Coordinates	Disease Overlap	Band
IKZF3	ENSG00000123411	IKZF4	$> 10^{-6}$	chr12:56201443..56601443	AA, PSO, T1D, VIT	12q13.2
IKZF3	ENSG00000204856	FAM216A	$> 10^{-6}$	chr12:110706169..111106169		12q24.11
IKZF3	ENSG00000111275	ALDH2	$> 10^{-6}$	chr12:112004691..112404691	AS, CEL, JIA, PBC, PSC, RA, T1D	12q24.12
IKZF3	ENSG00000089022	MAPKAPK5	$> 10^{-6}$	chr12:112079782..112479782	AS, CEL, JIA, PBC, PSC, RA, T1D	12q24.12
IKZF3	ENSG00000102580	DNAJC3	1.1e-05	chr13:96129393..96529393		13q32.1
IKZF3	ENSG00000185650	ZFP36L1	$> 10^{-6}$	chr14:69063190..69463190	CEL, CRO, JIA, MS, T1D	14q24.1
IKZF3	ENSG00000072110	ACTN1	4e-06	chr14:69246157..69646157	CEL, CRO, JIA, MS, T1D	14q24.1
IKZF3	ENSG00000100599	RIN3	2.3e-05	chr14:92780118..93180118		14q32.12
IKZF3	ENSG00000100811	YY1	7e-06	chr14:100504635..100904635		14q32.2
IKZF3	ENSG00000103811	CTSH	$> 10^{-6}$	chr15:79041916..79441916	MS, NAR, T1D	15q25.1
IKZF3	ENSG00000179583	CIITA	$> 10^{-6}$	chr16:10771055..11171055	CEL, MS, PBC, PSO, T1D	16p13.13
IKZF3	ENSG00000182108	DEXI	$> 10^{-6}$	chr16:10836317..11236317	CEL, MS, PBC, PSO, T1D	16p13.13
IKZF3	ENSG00000168488	ATXN2L	6e-06	chr16:28634356..29034356	AS, CRO, T1D	16p11.2
IKZF3	ENSG00000184517	ZFP1	2e-06	chr16:74982390..75382390	T1D	16q23.1
IKZF3	ENSG00000198931	APRT	1.2e-05	chr16:88678352..89078352		16q24.2
IKZF3	ENSG00000141012	GALNS	1.7e-05	chr16:88723378..89123378		16q24.3
IKZF3	ENSG00000185722	ANKFY1	$> 10^{-6}$	chr17:3967274..4367274		17p13.2
IKZF3	ENSG00000141753	IGFBP4	$> 10^{-6}$	chr17:38399702..38799702	T1D	17q21.1
IKZF3	ENSG00000108465	CDK5RAP3	3e-06	chr17:45845176..46245176	AS, MS	17q21.32
IKZF3	ENSG00000150637	CD226	1.4e-05	chr18:67429039..67829039	PSC, RA, T1D, UC	18q22.2
IKZF3	ENSG00000105655	ISYNA1	6e-06	chr19:18349111..18749111	MS	19p13.11
IKZF3	ENSG00000105700	KXD1	$> 10^{-6}$	chr19:18468572..18868572		19p13.11
IKZF3	ENSG00000105281	SLC1A5	1e-06	chr19:47091851..47491851	PSC, T1D, UC	19q13.32
IKZF3	ENSG00000042753	AP2S1	$> 10^{-6}$	chr19:47154249..47554249	PSC, T1D	19q13.32
IKZF3	ENSG00000087074	PPP1R15A	2e-06	chr19:49175649..49575649	CRO, T1D	19q13.33
IKZF3	ENSG00000104805	NUCB1	1e-06	chr19:49203307..49603307	CRO, T1D	19q13.33
IKZF3	ENSG00000160190	SLC37A1	$> 10^{-6}$	chr21:43716118..44116118	CEL, RA, T1D	21q22.3
IKZF3	ENSG00000185339	TCN2	$> 10^{-6}$	chr22:30802825..31202825		22q12.2
IKZF3	ENSG00000128311	TST	$> 10^{-6}$	chr22:37215681..37615681	JIA, T1D	22q12.3
IKZF3	ENSG00000100385	IL2RB	$> 10^{-6}$	chr22:37371094..37771094	JIA, T1D	22q12.3

Continued on next page

Supplementary Table 3 – continued from previous page

Transcription Factor	Ensembl ID	Name	$P_{.empirical}$	Coordinates	Disease Overlap	Band
IKZF3	ENSG00000100055	CYTH4	$> 10^{-6}$	chr22:37478068..37878068	JIA, T1D	22q12.3
IKZF3	ENSG00000128268	MGAT3	4e-06	chr22:39653349..40053349	CRO, PBC, UC	22q13.1
IKZF3	ENSG00000130826	DKC1	$> 10^{-6}$	chrX:153791031..154191031	T1D	Xq28
IKZF3	ENSG00000130830	MPP1	$> 10^{-6}$	chrX:153849282..154249282	T1D	Xq28
BATF	ENSG00000020633	RUNX3	0.00017	chr1:25091612..25491612	AS, PSO	1p36.11
BATF	ENSG00000236887		2e-06	chr1:113541447..113941447	ATD, CRO, JIA, RA, SLE, T1D	1p13.2
BATF	ENSG00000162889	MAPKAPK2	$> 10^{-6}$	chr1:206658289..207058289	CRO, SLE, T1D, UC	1q32.1
BATF	ENSG00000203705	TATDN3	0.00018	chr1:212765170..213165170		1q32.3
BATF	ENSG00000152291	TGOLN2	$> 10^{-6}$	chr2:85355548..85755548		2p11.2
BATF	ENSG00000144455	SUMF1	1e-06	chr3:3742498..4708965		3p26.2
BATF	ENSG00000175857	GAPT	0.00014	chr5:57587262..57987262		5q11.2
BATF	ENSG00000241685	ARPC1A	0.00022	chr7:98723521..99123521	CRO, UC	7q22.1
BATF	ENSG00000197157	SND1	$> 10^{-6}$	chr7:127092234..127732661		7q31.33
BATF	ENSG00000107249	GLIS3	$> 10^{-6}$	chr9:3824127..4548392	T1D	9p24.2
BATF	ENSG00000184293	CLECL1	$> 10^{-6}$	chr12:9685895..10085895	MS, T1D	12p13.31
BATF	ENSG00000111275	ALDH2	$> 10^{-6}$	chr12:112004691..112404691	AS, CEL, JIA, PBC, PSC, RA, T1D	12q24.12
BATF	ENSG00000152520	PAN3	0.00011	chr13:28512643..28912643		13q12.2
BATF	ENSG00000185650	ZFP36L1	$> 10^{-6}$	chr14:69063190..69463190	CEL, CRO, JIA, MS, T1D	14q24.1
BATF	ENSG00000170291	ELP5	0.00019	chr17:6954735..7354735		17p13.1
BATF	ENSG00000090339	ICAM1	0.00023	chr19:10181511..10581511	AS, CRO, JIA, MS, PBC, PSO, RA, T1D, UC	19p13.2
BATF	ENSG00000160190	SLC37A1	$> 10^{-6}$	chr21:43716118..44116118	CEL, RA, T1D	21q22.3
BATF	ENSG00000099995	SF3A1	$> 10^{-6}$	chr22:30552936..30952936	CRO, T1D, UC	22q12.2
BATF	ENSG00000128311	TST	$> 10^{-6}$	chr22:37215681..37615681	JIA, T1D	22q12.3
ESRRA	ENSG00000143321	HDGF	$> 10^{-6}$	chr1:156536717..156936717		1q23.1
ESRRA	ENSG00000143479	DYRK3	1e-06	chr1:206608881..207008881	CRO, SLE, T1D, UC	1q32.1
ESRRA	ENSG00000153551	CMTM7	6e-06	chr3:32233163..32633163		3p22.3
ESRRA	ENSG00000186106	ANKRD46	1.9e-05	chr8:101372012..101772012		8q22.2
ESRRA	ENSG00000164761	TNFRSF11B	6.1e-05	chr8:119764439..120164439		8q24.12
ESRRA	ENSG00000134453	RBM17	$> 10^{-6}$	chr10:5930950..6330950	AA, ATD, CRO, JIA, MS, PSC, RA, T1D	10p15.1
ESRRA	ENSG00000171206	TRIM8	3.5e-05	chr10:104204253..104604253		10q24.32

Continued on next page

Supplementary Table 3 – continued from previous page

Transcription Factor	Ensembl ID	Name	$P_{.empirical}$	Coordinates	Disease Overlap	Band
ESRRA	ENSG00000110651	CD81	$> 10^{-6}$	chr11:2197407..2597407	T1D	11p15.5
ESRRA	ENSG00000213619	NDUFS3	5.1e-05	chr11:47386888..47786888	MS	11p11.2
ESRRA	ENSG00000123444	KBTBD4	8.2e-05	chr11:47400567..47800567	MS	11p11.2
ESRRA	ENSG00000069493	CLEC2D	1e-06	chr12:9617565..10017565	MS, T1D	12p13.31
ESRRA	ENSG00000110848	CD69	2e-06	chr12:9713497..10113497	MS, T1D	12p13.31
ESRRA	ENSG00000062485	CS	7.1e-05	chr12:56494176..56894176	AA, PSO, T1D	12q13.2
ESRRA	ENSG00000122986	HVCN1	$> 10^{-6}$	chr12:110942755..111342755		12q24.11
ESRRA	ENSG00000089248	ERP29	$> 10^{-6}$	chr12:112251120..112651120	AS, CEL, JIA, PBC, PSC, RA, T1D	12q24.12
ESRRA	ENSG00000102580	DNAJC3	1.5e-05	chr13:96129393..96529393		13q32.1
ESRRA	ENSG00000100605	ITPK1	3.6e-05	chr14:93382665..93782665		14q32.12
ESRRA	ENSG00000168488	ATXN2L	5e-06	chr16:28634356..29034356	AS, CRO, T1D	16p11.2
ESRRA	ENSG00000153774	CFDP1	2.3e-05	chr16:75267383..75667383	T1D	16q23.1
ESRRA	ENSG00000198931	APRT	5e-06	chr16:88678352..89078352		16q24.2
ESRRA	ENSG00000185722	ANKFY1	$> 10^{-6}$	chr17:3967274..4367274		17p13.2
ESRRA	ENSG00000161395	PGAP3	2e-06	chr17:37653050..38053050	CRO, MS, PBC, RA, T1D, UC	17q12
ESRRA	ENSG00000141753	IGFBP4	$> 10^{-6}$	chr17:38399702..38799702	T1D	17q21.1
ESRRA	ENSG00000161654	LSM12	7.1e-05	chr17:41944987..42344987		17q21.31
ESRRA	ENSG00000167807		3.6e-05	chr19:10226685..10626685	AS, CRO, JIA, MS, PBC, PSO, RA, T1D, UC	19p13.2
ESRRA	ENSG00000076662	ICAM3	3.9e-05	chr19:10250499..10650499	AS, CRO, JIA, MS, PBC, PSO, RA, T1D, UC	19p13.2
ESRRA	ENSG00000105281	SLC1A5	$> 10^{-6}$	chr19:47091851..47491851	PSC, T1D, UC	19q13.32
ESRRA	ENSG00000198053	SIRPA	1.3e-05	chr20:1675154..2075154	T1D	20p13
ESRRA	ENSG00000160190	SLC37A1	$> 10^{-6}$	chr21:43716118..44116118	CEL, RA, T1D	21q22.3

Supplementary Table 3: Genes with significant $mean(-log(p_{T1D}))$ identified from enriched gene sets. Positions are given with respect to GRCh37, Ensembl ID's refer to release 75 of Ensembl. Alopecia Areata (AA), Ankylosing Spondylitis (AS) ATD - Autoimmune thyroid disease (ATD), Celiac disease (CEL), Crohn's disease (CD), Juvenile Idiopathic Arthritis (JIA), Multiple Sclerosis (MS), Narcolepsy (NAR), Primary Biliary Cirrhosis (PBC), Primary Sclerosing Cholangitis (PSC), Psoriasis (PSO), Rheumatoid Arthritis (RA), Sjogren's syndrome (SJO), Systemic Lupus Erythematosus (SLE), Ulcerative Colitis (UC), Vitiligo (VIT)
